# Systemic Ozone Therapy May Improve the Perception of Quality of Life: Ozonized Glucose Solution 5% Extends Reach to Patients Ineligible for Blood Infusion

**DOI:** 10.7759/cureus.64629

**Published:** 2024-07-16

**Authors:** Mauro Martinelli, Daniele Romanello

**Affiliations:** 1 Department of Biomedical Sciences, Ozone Therapy Unit, Ospedale San Pietro Fatebenefratelli, Roma, ITA; 2 Department of Internal Medicine, Ospedale San Pietro Fatebenefratelli, Roma, ITA

**Keywords:** heath-related quality of life, complementary treatments, ozonized glucose solution, quality of life (qol), oxygen-ozone therapy, sf36, ozone therapy

## Abstract

Ozone therapy is a complementary treatment that has gained popularity due to its safety and wide range of applications. Systemic ozone therapy involves withdrawing 100 to 200 ml of blood, treating it with an oxygen-ozone mixture, and then reinfusing it. This process requires large-caliber venous access, which can be a limitation. To overcome this, alternative administration methods have been explored, including the use of ozonized solutions. The aim of this study is to evaluate the clinical effects of systemic ozone therapy on the perception of quality of life and to analyze the outcomes of different administration methods. Three groups of patients were treated: one group received classical systemic ozone therapy, another received ozone therapy via intravenous infusion of a 5% glucose solution, and the third group alternated between the two methods. The results showed an improvement in perceived quality of life in all groups, regardless of the method used. Thus, systemic ozone therapy showed efficacy in improving the perception of quality of life in our group. Moreover, intravenous infusion of a 5% glucose solution has made it possible to treat patients who could not be treated with the classical method, achieving similar results.

## Introduction

Ozone therapy, a complementary treatment widely employed in common medical practice [1], has gained popularity due to its safety profile, rendering it suitable for broad application in conjunction with conventional therapies across a wide spectrum of diseases [2]. While local ozone therapy via direct injection enjoys widespread use, extracorporeal blood ozonation, also known as "great auto emo infusion" (GAEI), stands out as the most extensively studied method for systemic ozone therapy (SOT) administration [3]. This procedure involves withdrawing a precise volume of blood into an O3-resistant blood collection bag, to which 100-200 ml of an oxygen-ozone mixture at a specified concentration (in accordance with established protocols) is added. Following gentle mixing, the ozonated blood is reintroduced into the patient's circulation [4]. However, a notable challenge of this approach lies in the necessity of peripheral vein puncture with a large gauge needle (minimum 19 G) for blood collection. To address this limitation, alternative administration routes such as saline solution, glucose solution, or distilled water have been explored. Ozonized saline solution (0.9%) is a common alternative to GAEI in Russian medical practice [5]. Nevertheless, concerns have been raised regarding the potential formation of hypochlorous acid and other chlorine compounds [6-8]. Furthermore, direct infusion of distilled water can lead to osmotic damage due to its hypoosmolarity. An emerging alternative is ozonized glucose solution at a 5% concentration (OGS5%). This presents a safer option, as it is iso-osmolar (278 mOsm/L of 5% glucose compared to 275-295 mOsm/L of 0.9% saline solution) and contains only water and glucose. Recent investigations into the formation of toxic compounds from the OGS5% revealed minimal and essentially negligible chemical changes following ozone infusion [9]. Despite the documented efficacy of GAEI, there remains a dearth of literature on the clinically significant effects of OGS5%. The perspective of using this method is to expand the pool of patients who can undergo SOT, overcoming the limitations associated with the procedure required to perform GAEI. The objective of this study is to evaluate the clinical efficacy of SOT in improving the perception of quality of life (QOL) in patients with various medical conditions and to evaluate the subgroups based on the method of administration (OGS5%, GAEI, and both).

## Materials and methods

This is a retrospective observational study on 218 patients who were selected from the Ozone Therapy Center at San Pietro Fatebenefratelli Hospital in Rome, between May 2012 and December 2016, regardless of the disease being treated. The ethics committee has acknowledged the data analysis for the purpose of the retrospective study. All the patients were outpatients and naive to ozone therapy. Inclusion and exclusion criteria are listed in Table 1.

**Table 1:** Inclusion and exclusion criteria. All the exclusion criteria are common to the three groups. We excluded all the patients from the study who were not able to perform systemic ozone therapy as scheduled.

Inclusion criteria	Exclusion criteria
All patients requiring systemic ozone therapy	Anemia
	G6PDH deficit
	Impossibility to reach a venous access

We identify three groups. Each patient's venous status was evaluated, and when possible, the GAEI was the therapy of choice (they were assigned to Group 1). Patients with a reduced venous pool that did not allow for adequate compliance with therapy were treated with OGS5% and assigned to Group 2. Patients with poor venous status that was idoneous for blood ozone therapy but not enough to ensure its availability for the duration of the therapy schedule (or to spare the venous pool for further therapies) were assigned to Group 3. Group 1 underwent only GAEI, Group 2 received only OGS5%, and Group 3 underwent nine sessions of GAEI and nine sessions of OGS5% alternatively. All patients underwent a cycle of 18 administrations in total receiving all the same amount of ozone, following this protocol: two administrations per week for six weeks, followed by one per week for the subsequent six weeks, totaling 18 administrations over 12 weeks. The therapy schedule is listed in Table 2.

**Table 2:** Therapy schedule. This table shows the therapy schedule. Group 1 underwent only great auto emo infusion (GAEI), Group 2 underwent only ozonized glucose solution at a 5% concentration (OGS5%), and Group 3 underwent both alternatively.

	1st session	2nd session	3rd to 9th session	10th to 18th session
O2-O3 mixture (ml)	180	200	200	200
Concentration (mcg/ml)	35	35	40	50
Ozone (mg)	6	7	8	10
Blood (ml)	200	200	200	200
Glucose solution 5% (ml)	250	250	250	250

The therapy was prepared according to "Nuova FIO" guidelines and good practice [10]. Blood was collected and mixed in appropriate bags with an O2-O3 mixture, and then gently shaken for two minutes. The glucose solution was prepared with the same precautions required for blood therapy. The O2-O3 mixture was added to the solution (commonly used in clinical practice) using a fine 27G needle, preceded by an antibacterial filter. The small diameter of the needle allowed for bubbling, facilitating the mixing of gas and glucose solution. The solution was then gently shaken for two minutes, and excess gas was removed to enable isobaric infusion. An ozone generator with a photometer was used.

Prior to initiating therapy (T0), all patients completed the Short-Form 36 (SF-36), a questionnaire assessing QOL [11]. The test is routinely administered before and after therapy to obtain a report for the purpose of the treatment cycle. We have incorporated this practice into standard clinical practice independently of research purposes. The questionnaire comprises 36 questions contributing to eight domains defining the patient's overall health status, regardless of the specific disease present: physical functioning (10 questions), social functioning (four questions), bodily pain (two questions), role limitations due to emotional problems (three questions), role limitations due to physical problems (four questions), general health perceptions (one question), mental health (five questions), and vitality (four questions). The final score can be adjusted in the range from 0 to 100, with higher scores indicating better QOL. The patients completed the test again after the therapy (T1). For the statistical analysis, we used the independent two-sample t-test with the open-source package 'scipy' for the Python programming language.

## Results

**Figure 1:**
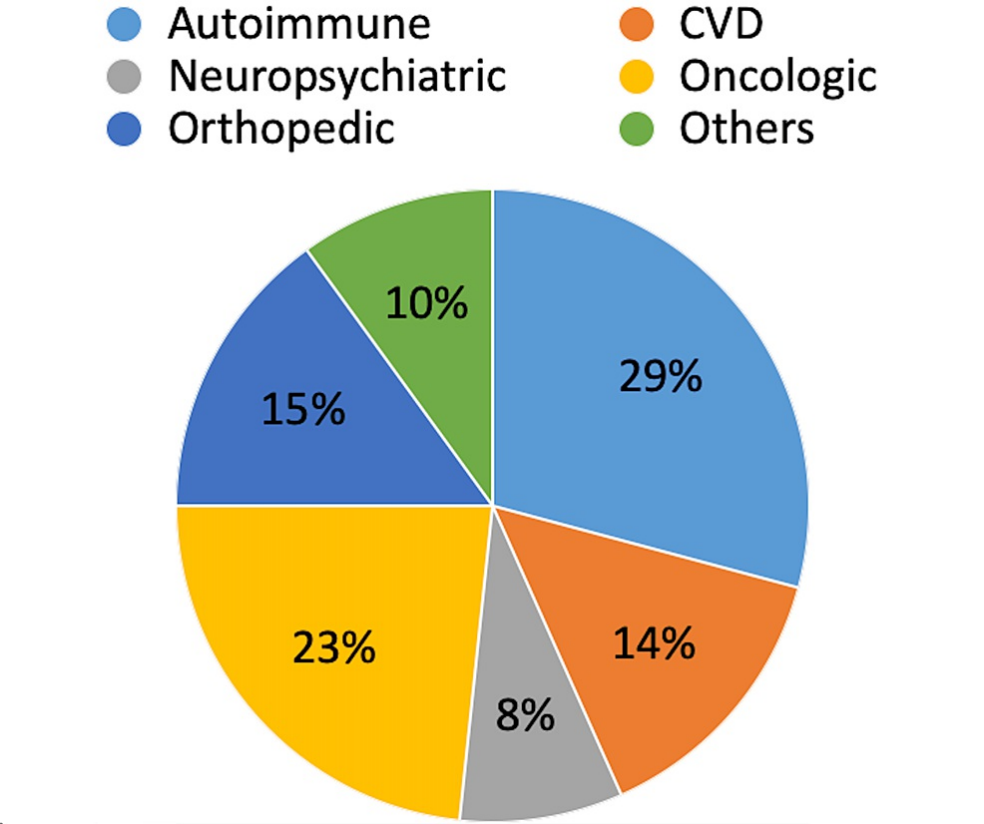
Percentage distribution of treated diseases. Distribution of treated diseases in the group that completed the therapeutic protocol. CVD: cardiovascular disease.

Only 120 patients completed the study: 41 in Group 1, 36 in Group 2, and 43 in Group 3 (Figure 1). It is noteworthy that none of the patients were excluded from the study due to lack of treatment continuity, and none experienced any adverse effects. No adverse effects were reported in the group who completed the study too.

**Figure 2:**
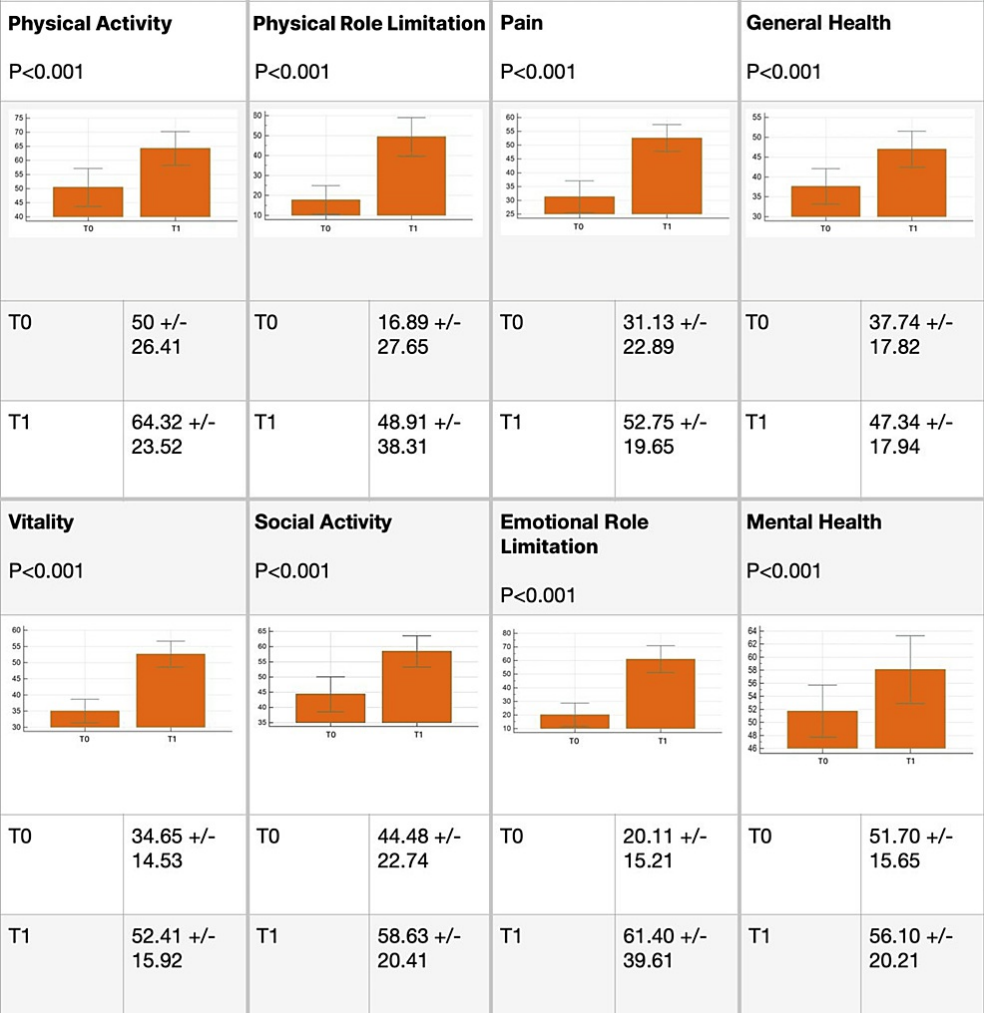
Global results. Global results in all sections of the Short-Form 36 (SF-36) before (T0) and after (T1) ozone therapy.

The exclusions were primarily attributed to discontinuation of therapy, missed sessions, changes in the therapeutic protocol, or the need to change the method of administration during the observation period. No one was excluded due to excess or lack of therapy efficacy, nor were there any reports of adverse reactions in any of the groups. The overall results are presented in Figure 2. Ozone therapy significantly improved the quality of life in all domains of the questionnaire.

**Figure 3:**
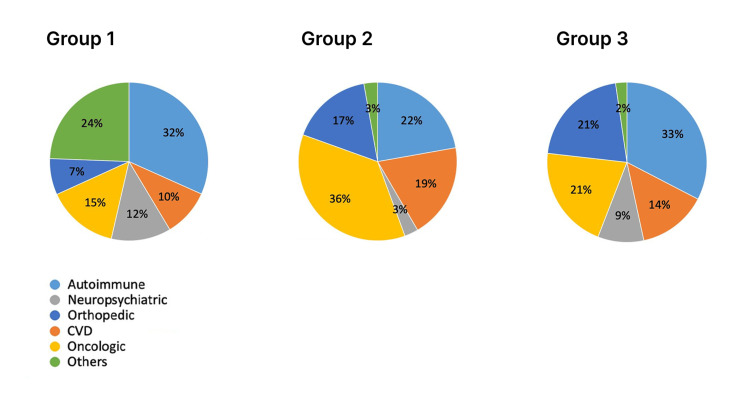
Differences between groups. The graphic shows the difference in pathologies between groups. CVD: cardiovascular disease.

The analysis of the groups revealed non-homogeneity in disease distribution when compared, as explained in the discussion (Figure 3), due to the criteria used for assignment to each group.

**Figure 4:**
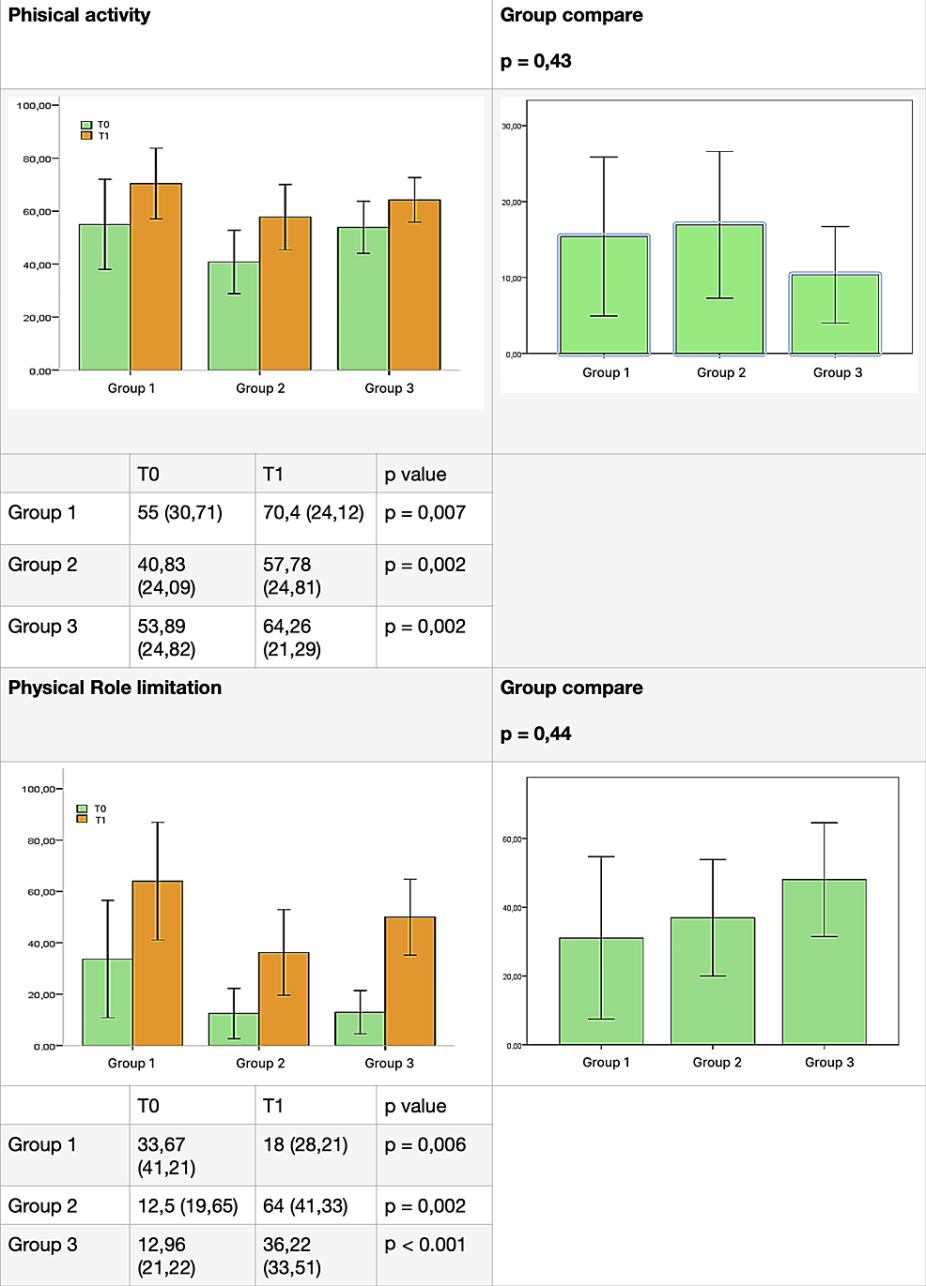
Comparative results between groups (part 1). Difference between groups before (T0) and after (T1) ozone therapy (physical activity and physical role limitation).

**Figure 5:**
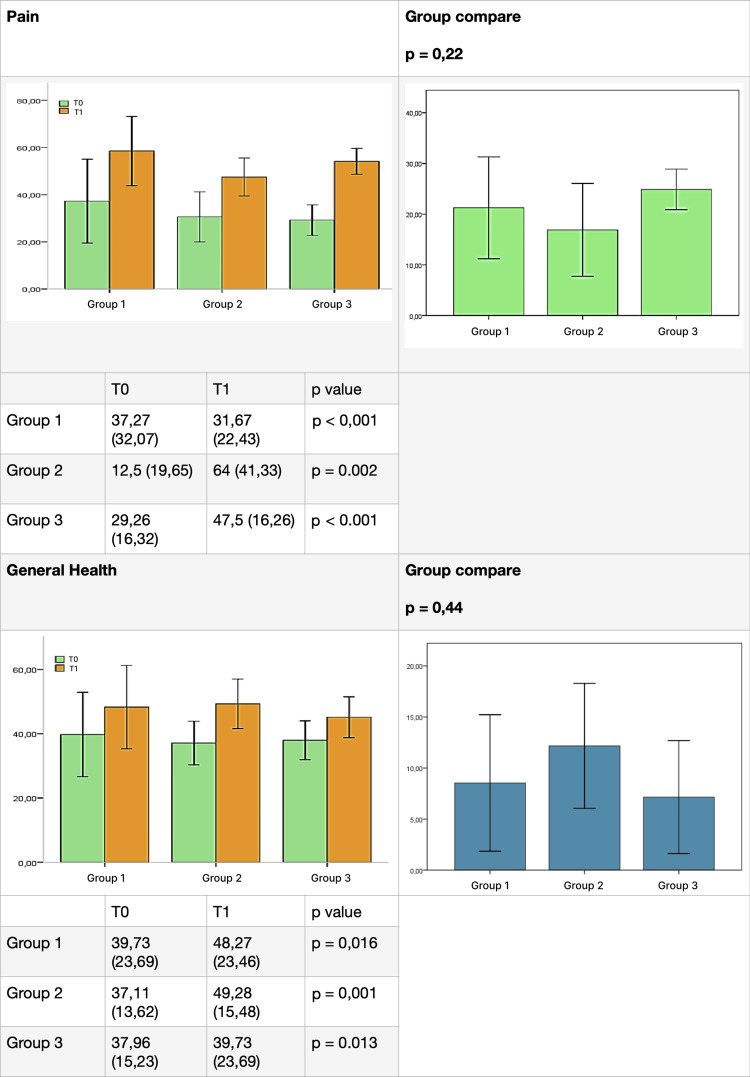
Comparative results between groups (part 2). Difference between groups before (T0) and after (T1) ozone therapy (pain and general health).

**Figure 6:**
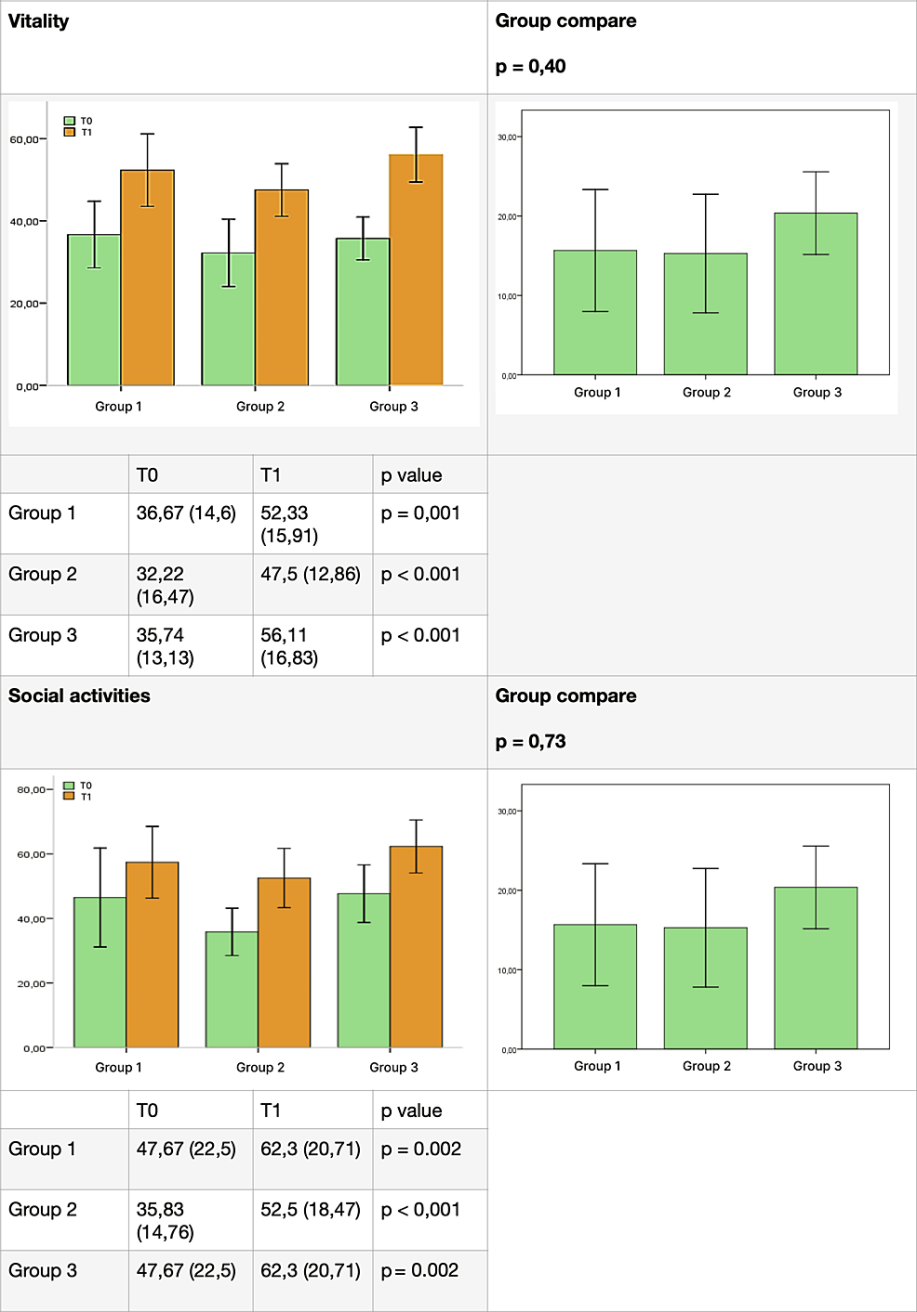
Comparative results between groups (part 3). Difference between groups before (T0) and after (T1) ozone therapy (vitality and social activity).

**Figure 7:**
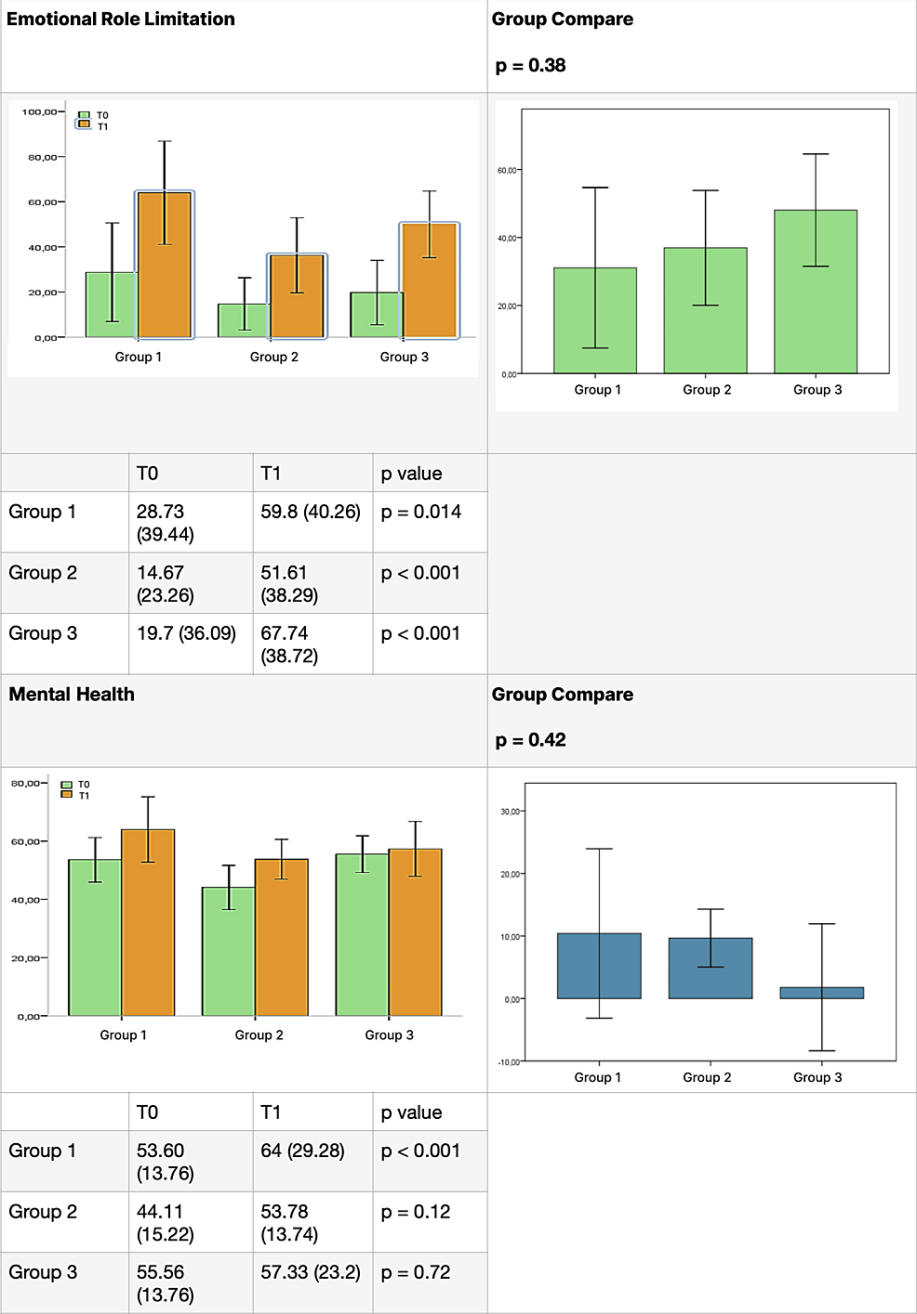
Comparative results between groups (part 4). Difference between groups before (T0) and after (T1) ozone therapy (emotional role limitation and mental health).

The results for each group, listed for each domain of the test before (T0) and after (T1) therapy, are presented in Figures 4-7. SOT demonstrated efficacy in almost all domains across all three groups, with a few exceptions. Group 1 did not show significant improvement in the "social activity" domain, while Groups 2 and 3 did not exhibit significant improvement in the "mental health" domain. Furthermore, the data did not reveal a statistically significant difference between the groups for each domain.

## Discussion

Ozone therapy is a safe complementary treatment that does not interfere with standard therapies. This study demonstrates how, when combined with standard treatments, ozone therapy can enhance the perceived QOL in patients with chronic diseases. The improvement is statistically significant across all domains of the SF-36 questionnaire, except for certain subgroup analyses. The observed enhancements in physical activity and physical role limitations are consistent with findings from previous studies on chronic fatigue [12-14]. The reduction in pain is unsurprising, given that ozone therapy is commonly used as an analgesic therapy, both systemically and topically [8]. It is evident that improvements in these domains can positively impact general health, vitality, and social activities. Increased energy, reduced limitations, and decreased pain contribute to greater self-wellness and activity levels. These factors have a profound effect on the psychological sphere, including emotional role limitations and mental health. Additionally, the literature suggests an antidepressant effect of ozone therapy [15,16]. Comparison of results between the various groups did not yield significant differences. This lack of significance is likely attributed to the small sample size, which may be further influenced by the diverse range of individual pathologies affecting the study participants. Group 1 is characterized by a wider spectrum of pathologies because the GAEI was proposed as the first option. In fact, Group 2 had a higher percentage of oncologic patients who are typically prone to having a poor vein asset due to long-lasting therapies such as chemotherapy.

This is the first study demonstrating the clinical effect of ozonized 5% glucose solution. This alternative method of administration is still under-researched. Some experts have expressed concerns about the safety of this procedure. One such concern is related to the "antioxidant buffer" provided by withdrawn blood when the oxygen-ozone mixture is infused. This buffer is generated by the physiological antioxidant activity of the blood itself, as well as by the peroxidation of lipids (resulting in lipid oxidation products) and proteins. Conversely, when the oxygen-ozone mixture is infused in a glucose solution, it dissolves and reacts with the solution itself before being infused into the patient. Although the total amount of ozone (and consequently its reactive compounds) is the same in both preparations, in the case of blood transfusion, it reacts with 200 ml of blood, whereas in the ozonized solution, it reacts with all the blood of the patient. Therefore, concerns regarding the inadequate "antioxidant buffer" appear inconsistent. As far as we know, the only concern regarding this procedure is determining the appropriate dose required for clinical efficacy and not for safety.

Besides the limitations of a retrospective study, the absence of a control group means that it is not possible to exclude the placebo effect. Other factors such as high dropout rates, group heterogeneity, and sample size also influence the significance of the study and should be taken into account when interpreting the results.

This study aims to provide promising insights into exploring this alternative method of administration, thereby expanding the possibility of treating patients with poor venous access, anemia, or the intention to preserve the venous pool. Furthermore, this alternative method of administration is also more cost-effective (as it does not require devices for blood withdrawal), faster, and easier to perform.

## Conclusions

This study, with the described limits, demonstrates the effectiveness of SOT in addition to standard therapy, in improving the perceived QOL in patients with various clinical conditions. While the data regarding treatment differences between GAEI and OGS5%, and both, did not reach significance, it is possible to affirm that SOT induced a statistically significant benefit in all three groups examined, regardless of the route of administration. Additionally, the introduction of this alternative method of administration could simplify SOT. However, it is important to note that measures involving perception can only reveal indirect results. Without a control group, the relationships between perceptions and QOL variables may be less robust. These findings need confirmation in a larger population, and the effects of the ozonized solution in improving the perception of QOL should be tested in prospective studies, blinded controlled studies, and blinded randomized controlled studies.
